# Editorial Note: Model of tumor dormancy/recurrence after short-term chemotherapy

**DOI:** 10.1371/journal.pone.0322161

**Published:** 2025-04-03

**Authors:** The PLOS One Editors

After publication of this article [1], concerns were raised that the Fig 2C left Actin panel in this article [1] appears similar to the Fig 1F Actin panel of a later article [2]. The first author confirmed that these Actin panels are the same image, and the Fig 2C left p21 panel in [1] and the Fig 1F HIF-1α panel in [2] are from the same experiment and were run on the same blot. They provided the underlying image data associated with the left Fig 2C blots (S1-S3 Files). The first author stated that in S3 File, blots were cut before staining for the Fig 2C left panel p21 in [1] and Fig 1F HIF-1α in [2].

Additionally, it was noted that in [1], the concentration of doxorubicin used is listed as 1 µM in the “Time Course- Cell Death Following Acute Chemotherapy Treatment” section of the article, whereas in the remainder of the article the doxorubicin concentration is listed as 1 µg/mL. The first author stated that 1 µg/mL is the correct doxorubicin concentration, and that 1 µM was a typographical error.

The *PLOS One* Editors issue this Editorial Note to inform readers of the above information and to relay the data provided.

## Supporting information

S1 File Data underlying Fig 2C left Actin panel.
(JPG)

S2 File Data underlying Fig 2C left panels showing p21 and Actin on the same blot.
The blot was first stained for p21 (red channel) before washing and staining for Actin (green channel).
(JPG)

S3 File Underlying data showing Fig 2C left p21 panel in [ 1 ] and Fig 1F HIF-1α panel in [ 2 ].
Top and middle: blots not used in [1], bottom left: Fig 1F HIF-1α in [2], bottom right: Fig 2C left p21 in [1]. The bottom blots were cut before detecting HIF-1α and p21.
(JPG)

## References

[1] Li S, Kennedy M, Payne S, Kennedy K, Seewaldt VL, Pizzo SV, et al. Model of tumor dormancy/recurrence after short-term chemotherapy. PLoS One. 2014;9(5): e98021. doi: 10.1371/journal.pone.0098021 24845582 PMC4028269

[2] Li S, Payne S, Wang F, Claus P, Su Z, Groth J, et al. Nuclear basic fibroblast growth factor regulates triple-negative breast cancer chemo-resistance. Breast Cancer Res. 2015;17(1):91. doi: 10.1186/s13058-015-0590-3 26141457 PMC4491247

