# Supplementary figures and images for: Editorial Note: Model of tumor dormancy/recurrence after short-term chemotherapy
Source: PLoS One. 2025 Apr 3;20(4):e0322161. doi: 10.1371/journal.pone.0322161 (PMC11967979; doi:10.1371/journal.pone.0322161)

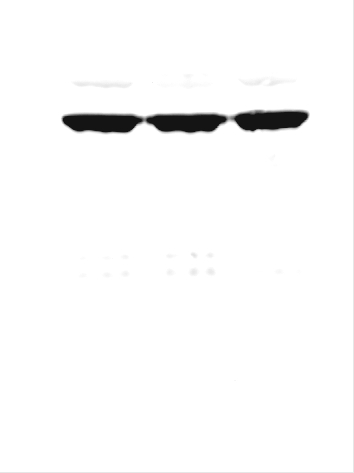

Supplement: S1 File — (JPG) [file pone.0322161.s001.jpg]

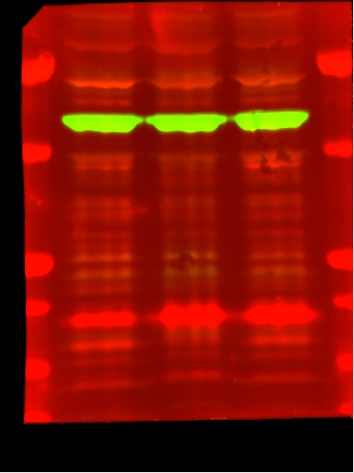

Supplement: S2 File — The blot was first stained for p21 (red channel) before washing and staining for Actin (green channel). (JPG) [file pone.0322161.s002.jpg]

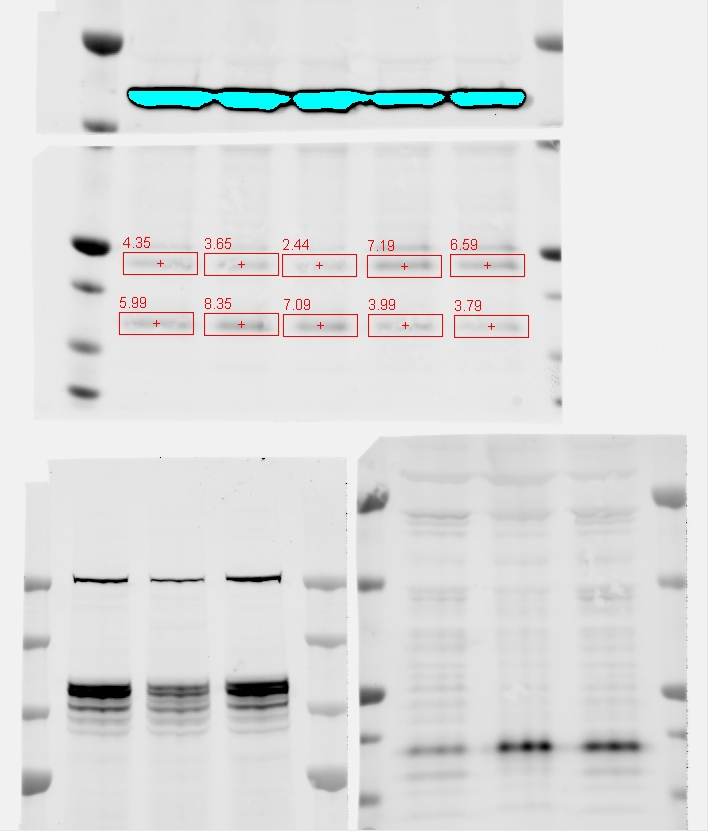

Supplement: S3 File — Top and middle: blots not used in [1], bottom left: Fig 1F HIF-1α in [2], bottom right: Fig 2C left p21 in [1]. The bottom blots were cut before detecting HIF-1α and p21. (JPG) [file pone.0322161.s003.jpg]
